# Bending or breaking? Examining the relationship between psychological inflexibility, resilience, professional fulfilment and burnout among cancer care professionals in Ireland

**DOI:** 10.3389/fpsyg.2026.1830006

**Published:** 2026-05-21

**Authors:** Lynn Farrell, Amanda Kracen, Robert Fox, Lynda Corrigan, Scheryll Alken, Dearbhaile Collins

**Affiliations:** 1 Psychology, School of Business and Social Sciences, National College of Ireland, Dublin, Ireland; 2 Tallaght University Hospital, Dublin, Ireland; 3 Children’s Health Ireland at Crumlin, Dublin, Ireland; 4 Trinity St. James Cancer Institute, Dublin, Ireland; 5 University College Cork Cancer Centre, Cork University Hospital, Cork, Ireland

**Keywords:** burnout, cancer care, health care professionals, professional fulfilment, psychological flexibility, psychological inflexibility, resilience

## Abstract

**Objective:**

Healthcare professionals (HCPs) in cancer care face increasing challenges with demand for care outpacing workforce growth. Professional demands contribute to decreases in job satisfaction and increases in experiences of burnout, which have serious implications for patient care and staff wellbeing. It is, therefore, important to determine factors that may buffer against burnout. This study focused on psychological inflexibility and resilience as psychological characteristics that may predict dimensions of burnout and support professional fulfilment.

**Methods:**

We administered an anonymous self-report survey to HCPs in Ireland through professional networks. Structural Equation Modelling assessed how gender, career stage, resilience and psychological inflexibility related to dimensions of burnout and professional fulfilment among 179 cancer care HCPs in Ireland.

**Results:**

Psychological inflexibility predicted higher emotional exhaustion and depersonalization, while resilience predicted lower professional inefficacy and higher professional fulfilment. Women reported higher emotional exhaustion and lower professional fulfilment. Early career HCPs showed higher depersonalization.

**Conclusion:**

Our findings demonstrate a relationship between psychological inflexibility, resilience and key markers of personal and professional wellbeing. This suggests a potential pathway for comprehensive intervention; while waiting for adequate systemic change, promoting greater resilience and psychological flexibility may support cancer care HCPs in the workplace.

## Introduction

Cancer care is in crisis, as the demand for healthcare professionals (HCPs) outpaces the growing need for staff. By 2030 there will be an estimated shortage of 4.1 million healthcare workers in Europe (European Confederation of Independent Trade Unions, 2022). However, need and role demands continue to grow with increased cancer cases (World Health Organisation, 2004), treatment complexity (Popescu et al., 2014) and administrative burdens (see Amesz et al., 2023).

Burnout is recognised as a serious problem for cancer care HCPs (e.g., Banerjee et al., 2017; Corrigan et al., 2020; De Hert, 2020; Murali et al., 2018). Maslach and Leiter (2016) defined burnout as a psychological syndrome involving severe exhaustion (emotional exhaustion), detachment and cynicism (depersonalization), and ineffectiveness (professional inefficacy) influenced by exposure to workplace stressors. Burnout, considered a public health crisis (West et al., 2018), contributes to negative personal and professional outcomes, including increased absenteeism (Dyrbye et al., 2019), clinician turnover (Willard-Grace et al., 2019), decreased job satisfaction and work effort (Shanafelt et al., 2016), reduced safety and poorer quality healthcare for patients (Panagioti et al., 2018; Salyers et al., 2017; Williams et al., 2007) and medical errors (Shanafelt et al., 2010). Among HCPs, burnout is related to depression and anxiety (Papathanasiou, 2015), problems with sleep, memory and muscle pain (Peterson et al., 2008). Some studies have also indicated that burnout has increased across the past decade (Schenkel et al., 2025). This highlights how imperative it is in the medical community to prioritise and address HCPs’ wellbeing (Hlubocky et al., 2021a).

Rates of burnout and dissatisfaction with work-life balance among HCPs has increased post-pandemic (see Lapen et al., 2025). Pre-pandemic, burnout was identified in 45% of consultants and 20% of Specialist Registrars in cancer care in Ireland (Corrigan et al., 2020). Cancer care was subsequently negatively impacted by the COVID-19 pandemic and a cyber-attack on the Irish Health Service Executive (MSD Ireland, 2021; O'Reilly et al., 2023), exacerbating challenging workplace conditions. Work-related stressors such as inefficient processes, poor leadership, negative work climates and excessive workloads contribute to burnout among HCPs (see West et al., 2018; Tetzlaff et al., 2022). Burnout may also increase due to delayed epidemic-related stressors as well as more immediate stressors (see Hlubocky et al., 2021b).

Given its influence, it is important to determine factors that may buffer against dimensions of burnout. Psychological flexibility and resilience are promising psychological factors. Psychological flexibility has been defined and examined by numerous theoretical traditions in psychology with common components including acting in line with values or goals while flexibly adapting to individual contexts and potential interference (Cherry et al., 2021). The current research operates within an Acceptance and Commitment Therapy (ACT; Hayes et al., 1999) informed framework in its approach to understanding and measuring psychological flexibility/inflexibility. Within ACT, psychological flexibility is a key target and is defined as a construct that supports individuals to willingly experience the present moment and commit to values-driven behaviour, regardless of uncomfortable internal experiences (i.e., thoughts and feelings; Hayes et al., 2004). While psychological flexibility involves adapting to ever-changing contexts and internal experiences (Hayes et al., 2006), psychological inflexibility involves unhelpful fusion with undesirable thoughts/beliefs and avoidance-based behaviour to escape unwanted internal experiences (see Macri and Rogge, 2024). Previous research has demonstrated that psychological flexibility/inflexibility is significantly related to all three dimensions of burnout in the workplace (Noone and Hastings, 2011; Ortiz-Fune et al., 2020), intentions to leave (Mollet et al., 2025), job performance and satisfaction (Bond and Bunce, 2003) and a range of indicators of wellbeing (e.g., Hayes et al., 2006). Increased psychological flexibility and decreased psychological inflexibility have been shown to reduce burnout (Dawson and Golijani-Moghaddam, 2020; Economides et al., 2022; Howell and Demuynck, 2021; Macri and Rogge, 2024; Puolakanaho et al., 2020) and reduce absenteeism (Finnes et al., 2019).

ACT interventions targeting psychological flexibility/inflexibility have demonstrated positive effects with HCPs and medical students reducing psychological distress (Mediavilla et al., 2023; Prudenzi et al., 2021; Prudenzi et al., 2022), the cognitive weariness dimension of burnout (e.g., difficulty concentrating; Prudenzi et al., 2022) and stress (Ditton et al., 2023), and improving mental health, quality of life (Otared et al., 2021), wellbeing (Ditton et al., 2023) and work-related quality of life (Garner and Golijani-Moghaddam, 2021). Exploring the relationship between psychological inflexibility and key outcomes (burnout and professional fulfilment) among Irish HCPs in cancer care is beneficial to understand whether it is an important psychological characteristic to target in interventions for wellbeing and job satisfaction.

Psychological resilience may uniquely contribute to burnout and professional fulfilment. Resilience represents how one adapts to current life circumstances and copes with stress (Connor and Davidson, 2003). While similar to psychological flexibility, they are distinct concepts (Jo et al., 2024). Resilience may support HCPs in managing demanding workplace environments and roles (Robertson et al., 2016) and was correlated with lower anxiety, depression and burnout among nurses (Hu et al., 2020) and oncologists (e.g., Budisavljevic et al., 2023) during the pandemic. It is suggested to buffer against burnout by influencing levels of engagement, self-efficacy and personal accomplishment (Hlubocky et al., 2017). Increasing resilience is thought to be important during times of crisis or great stress (Killgore et al., 2020). Given HCP’s high stress working environments, it is important to evaluate how resilience relates to dimensions of burnout and professional fulfilment and whether it uniquely predicts these factors over and above psychological inflexibility.

Additionally, we examined how gender and career stage relate to dimensions of burnout and professional fulfilment as previous research is mixed. Burnout and decreased professional fulfilment have been found in women physicians (Amoafo et al., 2015; Jalili et al., 2021; Lyubarova et al., 2023) and early career, younger HCPs (Amoafo et al., 2015; Jalili et al., 2021; El-Menyar et al., 2021; Shalaby et al., 2023; Lim et al., 2010), particularly emotional exhaustion (e.g., Elbarazi et al., 2017). High levels of depersonalization have been found among men (e.g., Jalili et al., 2021) and younger HCPs (Lim et al., 2010). Other studies, however, have not found gender and age differences (see Kesarwani et al., 2020; Lazarides et al., 2021).

To assess the relationship among these factors, we measured dimensions of burnout (emotional exhaustion, depersonalization, professional inefficacy), professional fulfilment, psychological inflexibility and resilience among HCPs in cancer care in Ireland. We hypothesised that gender, career stage, resilience and psychological inflexibility would predict professional fulfilment and dimensions of burnout.

## Method

This study was approved by the institutional Research Ethics Committee.

### Participants

Participants were 218 cancer care HCPs in Ireland; 39 respondents were excluded for only completing the consent form or demographics, leaving a sample of 179 HCPs. Participants could skip questions as this was determined to be the more ethical and inclusive approach compared to forced responding; as such, analyses involve varying sample sizes.

Participants were women (57.5%, *n* = 103), men (41.9%, *n* = 75) and one participant (0.6%) did not provide a valid gender response so was excluded from gender comparisons. Participants’ mean age was 41.39 years (*SD* = 8.98; range 25–68 years). Participants were categorised as early career if aged 40 and under (51.7%; *n* = 92) and seasoned career if aged over 40 (48.3%; *n* = 86) in line with previous definitions of young/early career oncologists (e.g., Banerjee et al., 2017). The mean years since medical school graduation was 16.60 years (*SD* = 9.10). Table 1 displays sample characteristics including role, medical specialties, ethnicity and country of citizenship. Medical oncologists (30.06%, *n* = 52) comprised the majority; six participants did not report a specialty. Most participants endorsed a White ethnic background (*n* = 160, 89.88%) and Irish citizenship (*n* = 153, 85.96%; see Table 2).

**Table 1:** Participant’s current roles (*n* = 172), specialty (*n* = 173), ethnicity (*n* = 178) and country of citizenship (*n* = 179).

Participant characteristics	% (*n*)
Role	
Consultant	55.81 (96)
Locum Consultant	4.65 (8)
Fellow	4.07 (7)
Specialist Registrar	24.42 (42)
Registrar	9.88 (17)
Other (incl. Associate Specialist, Higher Specialist Training)	1.74 (3)
Specialty	
Gynaecological Oncology	3.5 (6)
Haematology	13.9 (24)
Medical Oncology	30.1 (52)
Paediatric Oncology/Haematology	2.9 (5)
Palliative Care	15.6 (27)
Radiation Oncology	11 (19)
Surgical Oncology	19.7 (34)
Other (incl. Histopathology, Urology, Clinical Genetics)	3.5 (6)
Ethnicity	
Asian	7.30 (13)
Black	1.12 (2)
White	89.88 (160)
Another ethnicity (including Arab, Asian of African descent)	1.69 (3)
Country of citizenship	
Ireland	85.47 (153)
Another country (e.g., UK, Sudan, Malayasia, Pakistan, Poland)	14.53 (26)

**Table 2:** Descriptive statistics of the current study.

Variable	Mean (95% CI)	Median	SD	Range
Emotional exhaustion	26.30 (24.51/28.09)	25.00	11.29	3–51
Depersonalisation	8.10 (7.78/9.04)	7.00	5.94	0–23
Professional inefficacy	11.06 (9.87/12.26)	8.00	7.51	1–37
Professional fulfilment	21.29 (20.58/21.99)	22.00	4.57	7–23
Psychological inflexibility	19.24 (17.98/20.50)	18.00	8.16	7–45
Resilience	8.14 (7.94/8.34)	8.00	1.29	5–10

95% CI = 95% confidence intervals; SD = standard deviation.

### Measures

#### Burnout

The Maslach Burnout Inventory Human Services Survey for Medical Personnel (MBI-HSS-MP; Maslach and Jackson, 2016), assesses emotional exhaustion (9-items), depersonalization (5-items) and professional inefficacy (8-items). Items were summed separately for each subscale; higher scores indicated higher burnout. The MBI-HSS-MP has previously shown acceptable reliability and validity (Lin et al., 2022).

#### Psychological inflexibility

Psychological inflexibility was assessed using the 7-item Acceptance and Action Questionnaire-II (AAQ-II; Bond et al., 2011). Higher scores indicate higher psychological inflexibility. It has shown good validity and reliability (Bond et al., 2011).

#### Professional fulfilment

The 6-item professional fulfilment subscale of the Stanford Physician Wellness Survey’s Professional Fulfilment Index (Trockel et al., 2018) was used. Higher scores represent higher professional fulfilment. It has previously demonstrated acceptable validity and reliability (Trockel et al., 2018).

#### Resilience

The two-item version of the Connor-Davidson Resilience Scale (CD-RISC2; Vaishnavi et al., 2007) was administered. Higher scores indicate better resilience. The scale has been shown to have satisfactory validity and reliability (Davidson, 2020; Vaishnavi et al., 2007).

### Procedure

Participants were recruited by email and text through purposive and snowball sampling; messages were sent to relevant participants and professional organizations. The survey ran from May – July 2023 and asked about HCPs’ personal and professional experiences. Participants completed the self-report measures through an anonymous online survey using a cross-sectional correlational design.

### Analytic plan

Structural equation modelling (SEM) was used to examine the relationships between psychological inflexibility and resilience predicting burnout (emotional exhaustion, depersonalisation, professional inefficacy) and professional fulfilment, while controlling for exogenous covariates (gender [coded as 0 = women, 1 = men] and career stage [coded as 0 = early career, 1 = seasoned career]). Before evaluating the structural model, it was necessary to establish the measurement model (Anderson and Gerbing, 1988), that is, specifying only the latent variables. Data were analysed in Mplus 8.2 (Muthén and Muthén, 2018), and the models were estimated using the robust maximum likelihood (MLR) estimator. Missing data were handled using the robust full information maximum likelihood procedure.

Standard guidelines were followed to determine model fit (Hu and Bentler, 1999), using several goodness-of-fit indices. A nonsignificant χ^2^ indicates excellent model fit; however, a significant result (*p* < 0.05) should not lead to rejection of the model (Tanaka, 1987). In addition, Comparative Fit Index (CFI; Bentler, 1990) and Tucker-Lewis Index (TLI; Tucker and Lewis, 1973) values ≥ 0.90 indicate adequate model fit; and Root Mean Square Error of Approximation (RMSEA; Steiger, 1990) and Standardised Root Mean Square Residual (SRMR; Jöreskog and Sörbom, 1981) values ≤ 0.08 indicate adequate model fit. Internal reliability was assessed using composite reliability (ρ_c_) with values ≥ 0.60 deemed acceptable (Bagozzi and Yi, 1988).

Second, the moderating effect of gender on the relationship between psychological inflexibility, resilience, and career stage, and each of the latent outcome variables (emotional exhaustion, depersonalisation, professional inefficacy, professional fulfilment) was examined through moderated SEM analyses, using the latent moderated structural equations approach (LMS; Klein and Moosbrugger, 2000). Although the model in the prior step (i.e., without the interaction effects) analysed each outcome simultaneously, we analysed each outcome separately when including the interaction terms (i.e., four models with one model per outcome). This carries a disadvantage of multiple testing; however, given the limited sample size, including all interaction terms simultaneously would arguably result in substantial loss of statistical power and potentially reduce confidence within the findings.

## Results

### Measurement model and internal reliability

The measurement model consisting of six latent variables (emotional exhaustion, depersonalisation, professional inefficacy, professional fulfilment, psychological inflexibility, resilience) demonstrated satisfactory statistical fit (χ^2^ [614] = 1016.74, *p* < 0.001; CFI = 0.872; TLI = 0.861; RMSEA = 0.063 [90% CI 0.056, 0.070]); (SRMR = 0.076) according to the RMSEA and SRMR; however, statistical fit was slightly below satisfactory according to the CFI and TLI. As such, local sources of model misfit were inspected. Following inspection of the modification indices, it was found that model misfit was largely due to substantial residual covariances between several items with one residual covariance between emotional exhaustion items and two residual covariances between psychological inflexibility items: emotional exhaustion items (“I feel emotionally drained from my work” with “I feel used up at the end of the workday,” residual correlation = 0.43); psychological inflexibility items (“my painful memories prevent me from having a fulfilling life” with “my painful experiences and memories make it difficult for me to live a life that I would value,” residual correlation = 0.61; and “it seems like most people are handling their lives better than I am” with “worries get in the way of my success,” residual correlation = 0.43). These residual covariances were believed to be substantively meaningful and, thus, the model was re-evaluated to include these residual covariances. After re-specification, the model demonstrated satisfactory fit to the data (χ^2^ [611] = 908.01, *p* < 0.001; CFI = 0.905; TLI = 0.897; RMSEA = 0.054 [90% CI 0.047, 0.062]); (SRMR = 0.076). All factor loadings were positive and significant (*p* < 0.001). See Supplementary Table 1 for individual factor loadings and inter-factor correlations. Composite reliability estimates for emotional exhaustion (*ρ*_c_ = 0.91), depersonalisation (*ρ*_c_ = 0.79), professional inefficacy (*ρ*_c_ = 0.83), professional fulfilment (*ρ*_c_ = 0.90), psychological inflexibility (*ρ*_c_ = 0.92), and resilience (*ρ*_c_ = 0.70) factors all demonstrated satisfactory internal reliability. Table 2 displays descriptive statistics for each of these six variables.

### Structural model

**Figure 1:**
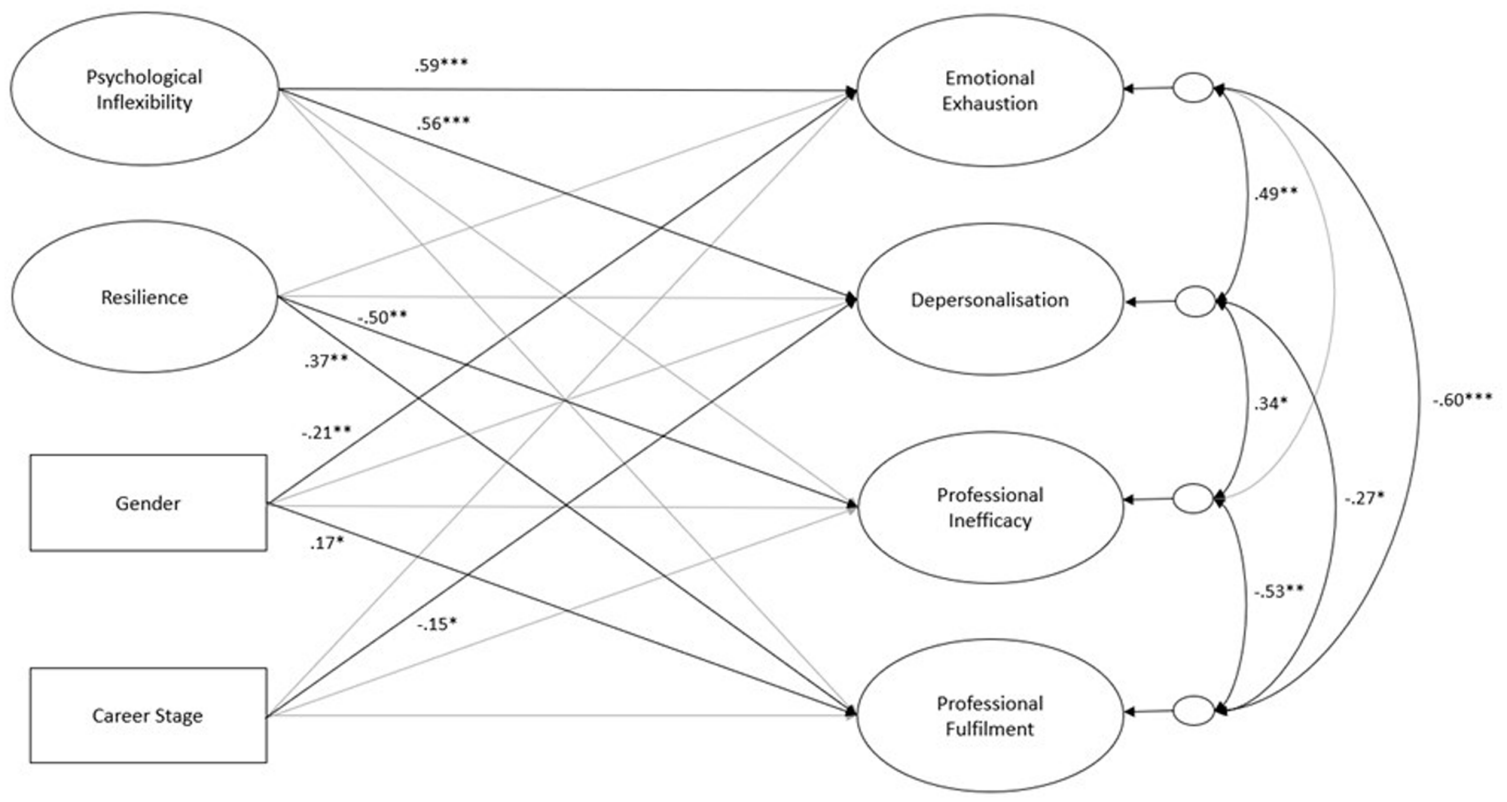
Structural model (standardised estimates) predicting emotional exhaustion, depersonalisation, professional inefficacy, and professional fulfilment. Note: Gender coded as 0 = woman, 1 = man; career stage coded as 0 = early career, 1 = seasoned career. Statistical significance: *p < 0.05; **p < 0.01; ***p < 0.001.

The statistical fit of the SEM model (see Figure 1) was mixed (χ^2^[677] = 1043.83, *p* < 0.001; CFI = 0.887; TLI = 0.877; RMSEA = 0.058 [90% CI 0.051, 0.065]); (SRMR = 0.079) with the absolute fit indices suggesting satisfactory fit to the data and the incremental fit indices falling below the desired conventional thresholds. Regarding variance, the model explained 42.5% of the variance in emotional exhaustion, 41.2% in depersonalisation, 32.9% in professional inefficacy, and 23.5% in professional fulfilment.

Psychological inflexibility was associated with emotional exhaustion (*β* = 0.59, *p* < 0.001) and depersonalisation (*β* = 0.56, *p* < 0.001); resilience was associated with lower levels of professional inefficacy (*β* = −0.50, *p* = 0.004) and higher levels of professional fulfilment (*β* = 0.37, *p* = 0.001); early career stage was associated with increased depersonalisation (*β* = −0.15, *p* = 0.047); and lastly, women were more likely to experience emotional exhaustion (*β* = −0.21, *p* = 0.003), whereas men were more likely to experience professional fulfilment (*β* = 0.17, *p* = 0.028). See Table 3 for full details.

**Table 3:** Standardised and unstandardised parameter estimates for the structural equation model predicting emotional exhaustion, depersonalisation, professional inefficacy, and professional fulfilment.

Predictors	Emotional exhaustion		Depersonalisation		Professional inefficacy		Professional fulfilment	
	B (SE)	β (SE)	B (SE)	β (SE)	B (SE)	β (SE)	B (SE)	β (SE)
Psychological inflexibility	0.78*** (0.15)	0.59 (0.08)	0.45*** (0.12)	0.56 (0.10)	0.08 (0.08)	0.13 (0.12)	−0.11 (0.09)	−0.14 (0.11)
Resilience	−0.11 (0.19)	−0.05 (0.09)	−0.13 (0.15)	−0.10 (0.12)	−0.47** (0.16)	−0.50 (0.11)	0.46** (0.14)	0.37 (0.10)
Gender^a^	−0.51** (0.17)	−0.21 (0.07)	0.12 (0.11)	0.08 (0.07)	−0.02 (0.09)	−0.02 (0.08)	0.25* (0.11)	0.17 (0.07)
Career stage	−0.16 (0.17)	−0.07 (0.07)	−0.22* (0.11)	−0.15 (0.07)	0.03 (0.08)	0.02 (0.08)	0.00 (0.12)	0.00 (0.08)

B = unstandardised estimates; β = standardised estimates; SE = standard error; ^a^gender coded as 0 = woman, 1 = man. Statistical significance: *p < 0.05; **p < 0.01; ***p < 0.001.

### Moderated SEM

Four moderated SEM models were examined to determine the moderating effect of gender on the relationship between each predictor variable (psychological inflexibility, resilience, career stage), and each outcome (emotional exhaustion, depersonalisation, professional inefficacy, professional fulfilment). Gender produced a significant interaction on the relationship between psychological inflexibility and professional inefficacy (*β* = 0.25, *p* = 0.037). However, when this effect was probed, no relationship was found to exist between psychological inflexibility and professional inefficacy for women (*β* = −0.07, *p* = 0.618) or men (*β* = 0.18, *p* = 0.124). No other significant interaction was found (see Supplementary Table 2 for all conditional and interaction effects).

## Discussion

Burnout is a global public health crisis among physicians with intervention strategies needed (Chirico et al., 2021) including at the organizational level (Lapen et al., 2025). It is important to determine what psychological factors may influence HCPs’ wellbeing and whether certain groups (gender, career stage) may benefit from targeted interventions. This study examined how gender, career stage, resilience and psychological inflexibility related to dimensions of burnout and professional fulfilment among HCPs in cancer care in Ireland. While gender did not moderate results, women scored higher on emotional exhaustion and lower on professional fulfilment, compared to men. Early career HCPs, regardless of gender, showed higher depersonalization. This supports previous findings that women and younger, early career HCPs are at higher risk for these dimensions of burnout (Elbarazi et al., 2017; El-Menyar et al., 2021; Lim et al., 2010) and lower professional fulfilment among women (Lyubarova et al., 2023).

Consistent with previous research that psychological inflexibility and resilience are separate but related concepts (Jo et al., 2024), these two factors made unique predictions in our model. Psychological inflexibility predicted higher emotional exhaustion and depersonalization, while resilience predicted lower professional inefficacy and higher professional fulfilment. This expands on previous literature considering these factors separately (e.g., Budisavljevic et al., 2023; Prudenzi et al., 2022) and points to the potential for a comprehensive intervention approach.

### Practical implications

These findings suggest that HCPs in cancer care may benefit from increased resilience and reduced psychological inflexibility given their relationship to professional fulfilment and wellbeing – factors that are important to alleviate workforce shortages and poor health (Hlubocky et al., 2021a, 2021b; Lim et al., 2024). This has implications for how healthcare leadership and researchers intervene. Influencing dimensions of burnout may require targeting different psychological factors; psychological inflexibility appears to be a relevant correlate of emotional exhaustion and depersonalization, while resilience may be more relevant for professional efficacy. Our findings indicate that interventions to reduce psychological inflexibility and increase resilience may be useful for men and women, across career stages. Women would benefit from interventions targeting their reported higher emotional exhaustion and lower professional fulfilment, while depersonalization appears a particular concern for early career HCPs. All interventions should also acknowledge and address organizational culture to implement policies and practices that create supportive environments (see Lapen et al., 2025). As is increasingly clear from the literature, comprehensive interventions that address both structural and psychological components are needed to support the wellbeing of HCPs in cancer care (Erul et al., 2026). Our findings further support this call to action by highlighting further psychological factors to consider for specific burnout dimensions and professional fulfilment.

Psychological flexibility (see Gloster et al., 2017) and resilience (see Rakesh et al., 2017) are both malleable following targeted interventions. Promisingly, ACT-informed interventions have shown utility in reducing psychological inflexibility and increasing psychological flexibility and resilience (Macri and Rogge, 2024) including among HCPs (e.g., Bolderston et al., 2020). Thus, ACT may represent an effective theoretical framework for work-based interventions for HCPs. One approach to intervention could involve structured mentoring programs. Mentoring programs have previously improved personal and professional outcomes for medical doctors (e.g., Houchens et al., 2024; Winderbaum and Coventry, 2024) and can be bespoke to individual schedules.

### Limitations and future directions

As this study used a cross-sectional design, this precludes drawing causal/temporal inferences. While recruitment was comprehensive, it is difficult to gauge how representative the sample is without access to the total number of HCPs in cancer care; these numbers are not available in Ireland. We were unable to calculate our response rate as our denominator was unknown due to use of snowball sampling and lack of information regarding membership numbers in organization and communication channels utilised. This challenge has similarly been faced by other research on HCPs’ wellbeing (e.g., Erul et al., 2026; Schenkel et al., 2025). While participants were invited to take part in a national benchmarking survey, the possibility of selection bias remains where individuals with stronger views on wellbeing may have been more likely to participate. We captured a range of specialties and roles; however, our sample was majority White, recruited in the Republic of Ireland and consisted of people reporting binary genders only, all which limit preferred intersectional analyses. We anticipate that our findings generalise based on previous related research outside of Ireland, however, caution is warranted given the limited diversity of the sample and findings should be considered in context. Future research should seek larger, more diverse samples, examining additional social identities that may influence findings. Previous research has suggested discrimination and mistreatment by patients and visitors is more common for women and ethnic minority physicians and is associated with burnout (Dyrbye et al., 2022). However, Garcia et al. (2020) suggested ethnic minorities experience less burnout than non-Hispanic White physicians, although this was challenged by Cantor and Mouzon (2020) who encourage more complex research approaches. This highlights the importance of thorough, nuanced investigations to understand how to support cancer care HCPs’ unique experiences.

Age was chosen as a proxy for career stage in line with recommendations from the oncologists on the research team consistent with the European Society for Medical Oncology’s criteria of young oncologists (Banerjee et al., 2017) and related papers focused on young or junior HCPs (e.g., Ciammella et al., 2013). It also represented a more complete set of responses to base career stage classification on. This may, however, align more with age group categories (e.g., young HCPs vs. older HCPs) rather than career stage. Further research may explore a more direct means of categorising career stage to determine whether the findings are influenced, however, decisions vary across studies and contexts as to when early career ends.

The measurement model revealed residual correlations among some of the observed variables within the emotional exhaustion and psychological inflexibility constructs, suggesting that there might be issues with the construct validity of these measures. A possible reason for these correlations is that the items are too similar in their wording or are measuring overlapping aspects of the same construct. We addressed these by allowing the residuals to covary in our model; however, future research should further examine the cause behind these residual correlations and ways in which the measure can be improved. For example, through the removal of redundant items or rephrasing of similar items to improve the psychometric properties of these measures.

The AAQ-II and CD-RISC2 are commonly used measures when assessing psychological inflexibility and resilience, respectively, among HCPs. Their brevity (alongside good psychometric properties) was an important feature when targeting a population already managing heavy professional demands. This brevity may have constrained conceptual depth, however. Additionally, recent literature has begun to question the discriminant validity of the AAQ-II (e.g., Wolgast, 2014; though cf. Ruiz et al., 2024). Future research may, therefore, consider examining psychological inflexibility, flexibility and resilience using more comprehensive and, in the case of psychological inflexibility, multidimensional measures. This would allow for a more nuanced examination of what components of psychological inflexibility (e.g., cognitive fusion; Deledda et al., 2025) may be related to personal and professional wellbeing among HCPs. Measures of psychological flexibility could also be considered alongside psychological inflexibility measures as previous research has often focused on assessing psychological inflexibility via the AAQ-II (Macri and Rogge, 2024).

Recognising the importance of psychological inflexibility and resilience, a promising further direction is to develop ACT-based interventions for HCPs in cancer care (e.g., Bolderston et al., 2020). Future work should strive to integrate interventions without adding to the occupational burdens already faced by HCPs. Continued evaluation and comparison of interventions is crucial to determine which are most effective and whether effects maintain over time. Even 1-point changes in burnout scores can have meaningful positive effects on adverse outcomes (see West et al., 2016).

While our results advocate for individualised interventions that increase psychological flexibility and resilience, we recognise that individual-level interventions alone will not fully address challenges facing our healthcare workforce (see Erul et al., 2026; Lapen et al., 2025). System-based interventions are crucial for sustainable change (Yates, 2020). We strongly advocate for systemic changes that support greater wellbeing among HCPs.

## Conclusion

Among cancer care HCPs in Ireland, psychological inflexibility is related to emotional exhaustion and depersonalization, while resilience is related to lower professional inefficacy and higher professional fulfilment. Women experienced higher emotional exhaustion and lower professional fulfilment, while early career HCPs showed higher depersonalization. Our findings advocate for interventions to increase psychological flexibility and resilience. Both individual- and system-level interventions are necessary for increased effectiveness (Lapen et al., 2025). While waiting for adequate systemic interventions, reducing psychological inflexibility and promoting psychological flexibility and resilience may support cancer care HCPs in the workplace.

## Data Availability

The original contributions presented in the study are included in the article/Supplementary material, further inquiries can be directed to the corresponding authors.
