# Correction: Bending or breaking? Examining the relationship between psychological inflexibility, resilience, professional fulfilment and burnout among cancer care professionals in Ireland

**DOI:** 10.3389/fpsyg.2026.1894803

**Published:** 2026-06-23

**Authors:** Lynn Farrell, Amanda Kracen, Robert Fox, Lynda Corrigan, Scheryll Alken, Dearbhaile Collins

**Affiliations:** 1 Psychology, School of Business and Social Sciences, National College of Ireland, Dublin, Ireland; 2 Tallaght University Hospital, Dublin, Ireland; 3 Children's Health Ireland at Crumlin, Dublin, Ireland; 4 Trinity St. James Cancer Institute, Dublin, Ireland; 5 University College Cork Cancer Centre, Cork University Hospital, Cork, Ireland

**Keywords:** burnout, cancer care, health care professionals, professional fulfilment, psychological flexibility, psychological inflexibility, resilience

In the published article, there was a mistake in the section **Method**, *Measures*, Professional fulfilment description. The description in the original article said, “Mean scores of 3 or more indicate professional fulfilment.” However, response options ranged from 1-5 for each Professional Fulfilment item in the current study (indicating mean scores of 4 and above = professional fulfilment) and total scores, not mean scores, were used in the current analysis. This sentence should read as “Higher scores represent higher professional fulfilment.” This does not change the results or interpretation of the inferential analysis.

A correction has been made to the section **Method**, *Measures*, Professional fulfilment, Paragraph 3:

“Higher scores represent higher professional fulfilment.”

The original version of this article has been updated.

