# Supplementary material for: Bending or breaking? Examining the relationship between psychological inflexibility, resilience, professional fulfilment and burnout among cancer care professionals in Ireland
Source: Front Psychol. 2026 May 21;17:1830006. doi: 10.3389/fpsyg.2026.1830006 (PMC13233423; doi:10.3389/fpsyg.2026.1830006)
Supplement: Supplementary file 1 [file Supplementary_file_1.docx]

**Supplementary Table 1***Standardised factor loadings and factor correlations (and standard errors) for the measurement model.*

|  | Emotional exhaustion | Depersonalisation | Professional inefficacy | Professional fulfilment | Psychological inflexibility | Resilience |
| --- | --- | --- | --- | --- | --- | --- |
| ***Factor loadings*** |  |  |  |  |  |  |
| MBI_1 | .81 (.03) |  |  |  |  |  |
| MBI_2 | .78 (.03) |  |  |  |  |  |
| MBI_3 | .79 (.03) |  |  |  |  |  |
| MBI_6 | .63 (.06) |  |  |  |  |  |
| MBI_8 | .85 (.03) |  |  |  |  |  |
| MBI_13 | .69 (.05) |  |  |  |  |  |
| MBI_14 | .60 (.06) |  |  |  |  |  |
| MBI_16 | .61 (.06) |  |  |  |  |  |
| MBI_20 | .79 (.04) |  |  |  |  |  |
| MBI_5 |  | .51 (.08) |  |  |  |  |
| MBI_10 |  | .85 (.04) |  |  |  |  |
| MBI_11 |  | .83 (.05) |  |  |  |  |
| MBI_15 |  | .53 (.08) |  |  |  |  |
| MBI_22 |  | .50 (.07) |  |  |  |  |
| MBI_4 |  |  | .49 (.11) |  |  |  |
| MBI_7 |  |  | .54 (.10) |  |  |  |
| MBI_9 |  |  | .69 (.06) |  |  |  |
| MBI_12 |  |  | .53 (.07) |  |  |  |
| MBI_17 |  |  | .67 (.09) |  |  |  |
| MBI_18 |  |  | .64 (.07) |  |  |  |
| MBI_19 |  |  | .76 (.06) |  |  |  |
| MBI_21 |  |  | .59 (.06) |  |  |  |
| PFI_1 |  |  |  | .83 (.05) |  |  |
| PFI_2 |  |  |  | .84 (.04) |  |  |
| PFI_3 |  |  |  | .88 (.03) |  |  |
| PFI_4 |  |  |  | .63 (.05) |  |  |
| PFI_5 |  |  |  | .77 (.04) |  |  |
| PFI_6 |  |  |  | .71 (.05) |  |  |
| AAQ-II_1 |  |  |  |  | .67 (.06) |  |
| AAQ-II_2 |  |  |  |  | .88 (.03) |  |
| AAQ-II_3 |  |  |  |  | .88 (.03) |  |
| AAQ-II_4 |  |  |  |  | .79 (.04) |  |
| AAQ-II_5 |  |  |  |  | .84 (.03) |  |
| AAQ-II_6 |  |  |  |  | .78 (.04) |  |
| AAQ-II_7 |  |  |  |  | .71 (.05) |  |
| CD-RISC2_1 |  |  |  |  |  | .77 (.06) |
| CD-RISC2_2 |  |  |  |  |  | .70 (.07) |
| ***Factor correlations*** |  |  |  |  |  |  |
| Emotional exhaustion | 1 |  |  |  |  |  |
| Depersonalisation | .66 (.08) | 1 |  |  |  |  |
| Professional inefficacy | .39 (.09) | .47 (.09) | 1 |  |  |  |
| Professional fulfilment | -.64 (.06) | -.38 (.10) | -.63 (.08) | 1 |  |  |
| Psychological inflexibility | .63 (.07) | .62 (.07) | .37 (.09) | -.34 (.10) | 1 |  |
| Resilience | -.31 (.09) | -.40 (.11) | -.56 (.08) | .41 (.09) | -.49 (.08) | 1 |

Note: All factor loadings are statistically significant (p < .001); MBI = Maslach Burnout Inventory Human Services Survey for Medical Personnel; PFI = Stanford Physician Wellness Survey’s Professional Fulfilment Index; AAQ-II = Acceptance and Action Questionnaire-II; CD-RISC2 = Connor-Davidson Resilience Scale.

**Supplementary Table 2**

*Standardised and unstandardised parameter estimates for separate moderated structural equation models predicting emotional exhaustion, depersonalisation, professional inefficacy, and professional fulfilment.*

|  | Emotional exhaustion | |  | Depersonalisation | |  | Professional inefficacy | |  | Professional fulfilment | |
| --- | --- | --- | --- | --- | --- | --- | --- | --- | --- | --- | --- |
|  | B (SE) | β (SE) |  | B (SE) | β (SE) |  | B (SE) | β (SE) |  | B (SE) | β (SE) |
| Psychological inflexibility | .65*** (.17) | .48 (.12) |  | .48*** (.13) | .57 (.12) |  | -.04 (.09) | -.07 (.14) |  | -.02 (.11) | -.02 (.14) |
| Resilience | -.22 (.28) | -.10 (.13) |  | -.12 (.18) | -.10 (.14) |  | -.58** (.19) | -.60 (.15) |  | .61*** (.17) | .49 (.12) |
| Gender^a^ | -.53* (.24) | -.21 (.09) |  | -.01 (.16) | .00 (.11) |  | -.02 (.12) | -.02 (.11) |  | .13 (.16) | .09 (.11) |
| Career stage | -.15 (.25) | -.06 (.10) |  | -.34* (.15) | -.22 (.09) |  | .03 (.11) | .02 (.10) |  | -.10 (.16) | -.07 (.11) |
| Gender x psych inflexibility | .42 (.24) | .15 (.08) |  | .01 (.17) | .00 (.10) |  | .32* (.15) | .25 (.10) |  | -.17 (.19) | -.11 (.12) |
| Gender x resilience | .28 (.41) | .07 (.09) |  | .04 (.35) | .02 (.14) |  | .22 (.21) | .11 (.11) |  | -.27 (.24) | -.11 (.09) |
| Gender x career stage | .05 (.33) | .02 (.11) |  | .29 (.22) | .15 (.12) |  | .04 (.17) | .03 (.12) |  | .22 (.24) | .12 (.13) |

Note: B = unstandardised estimates; β = standardised estimates; SE = standard error; ^a^ = gender coded as 0 = woman, 1 = man; Psych inflexibility = psychological inflexibility.
Statistical significance: **p* < .05; ***p* < .01; ****p* < .001.
